# Supplementary material for: A Novel Colorimetric Sensor Array Coupled Multivariate Calibration Analysis for Predicting Freshness in Chicken Meat: A Comparison of Linear and Nonlinear Regression Algorithms
Source: Foods. 2023 Feb 7;12(4):720. doi: 10.3390/foods12040720 (PMC9955736; doi:10.3390/foods12040720)
Supplement: Supplementary file 1 [file foods-12-00720-s001.zip › foods-2175637-supplementary.pdf]

A Novel Colorimetric Sensor Array Coupled Multivariate Calibration Analysis for Predicting Freshness in Chicken Meat: A Comparison of Linear and Nonlinear Regression Algorithms

Wenhui Geng [1,†], Suleiman A. Haruna [1,2,†], Huanhuan Li [1,*], Hafizu Ibrahim Kademi [2] and Quansheng Chen [3,*]

[1] School of Food and Biological Engineering, Jiangsu University, Zhenjiang 212013, China
[2] Department of Food Science and Technology, Kano University of Science and Technology, Wudil, P.M.B 3244 Kano, Kano State, Nigeria
[3] College of Food and Biological Engineering, Jimei University, Xiamen 361021, China
* Correspondence: jiona1044@ujs.edu.cn (H.L.); qschen@ujs.edu.cn (Q.C.); Tel.: +86-511-88790318 (Q.C.); Fax: +86-511-88780201 (Q.C.)
† These authors contributed equally to this work.

The theory of Runs test

The residuals between the first several principal components of the spectral data and the second derivative of the first principal component were extracted by the principal component analysis (PCA). Then, by analyzing the relationship between the overall positive residuals (n_+), negative residuals (n_-) and the number of runs (u) the nonlinearity of CSA data were finally tested. *z* represents the randomness of testing CSA data. When $n_+ > 10$ and $n_- > 10$, the below approximations generated acceptable results:

$$\mu = \frac{2n_+n_-}{n_+ + n_-} + 1 \quad (1)$$

$$\sigma^2 = \frac{2n_+n_-(2n_+n_- - n_+ - n_-)}{(n_+ + n_-)^2(n_+ + n_- - 1)} \quad (2)$$

$$z = \frac{u - \mu + 0.5}{\sigma} \quad (3)$$

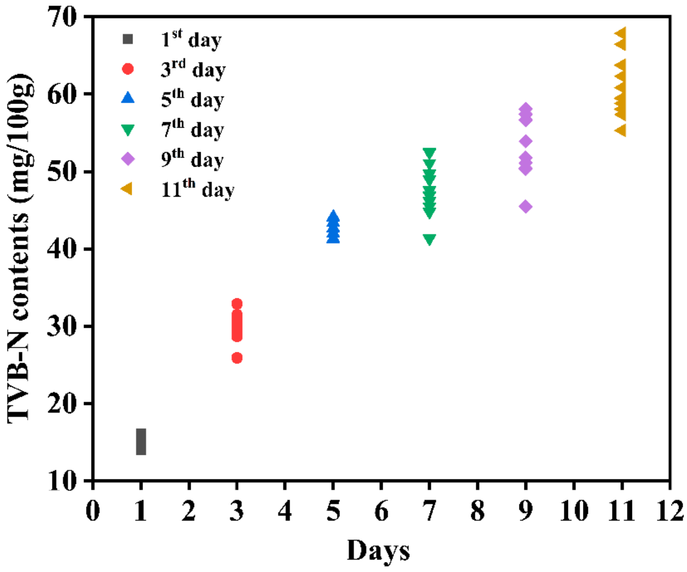

Figure S1. Scatter plot showing chemical reference measurements values of TVB-N in the chicken breast fillets during 11days.

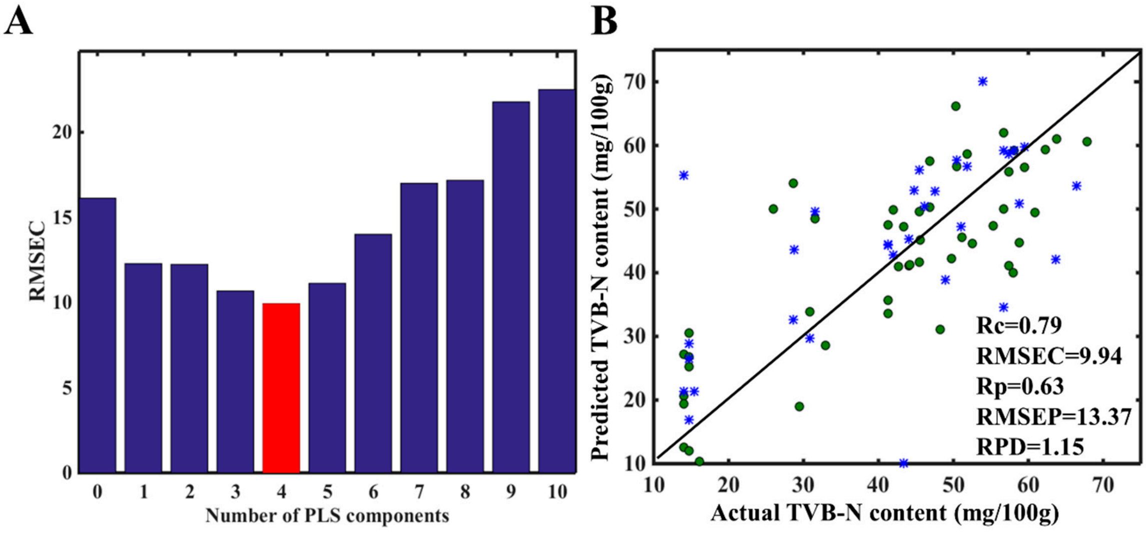

Figure S2. PLS (linear) performance based on (**A**) number of latent variables; and (**B**) scatter plot result of actual *vs.* predicted values for TVB-N content in a chicken meat sample.

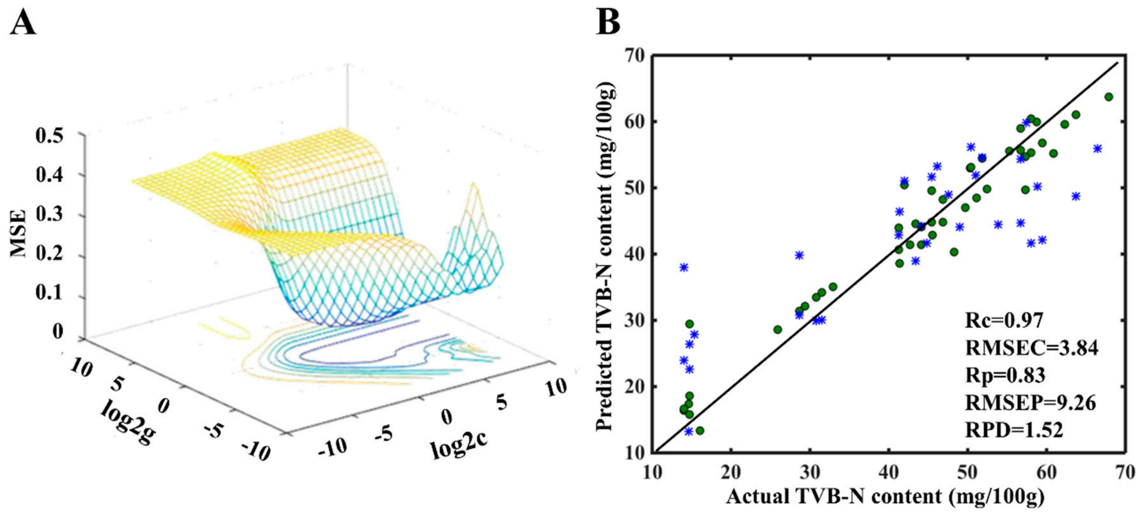

Figure S3. SVM (nonlinear) performance based on (**A**) optimization of SVM model parameters and (**B**) a scatter plot result of actual *vs.* predicted values for TVB-N content in a chicken meat sample.

Table S1. Statistics table of the chemical reference measurements values of TVB-N in the chicken breast fillets during 11days.

**Qverall**	**Minimum**	**Maximum**	**Mean**	**Standard deviation**	**Range**
TVB-N contents (mg/100g)	13.98	67.85	42.05	15.86	13.98-67.85

Table S2. Name and formula of chemoresponsive dyes used for fabricating CSA.

Number	Chemoresponsive Dyes	Formula
1	5,10,15,20-Tetraphenyl-21H,23H-porphine iron(III)chloride	$C_{44}H_{30}Cl_3FeN_4$
2	5,10,15,20-Tetraphenyl-21H,23H-porphine copper(II)chloride	$C_{44}H_{30}CuN_4$
3	5,10,15,20-Tetrakis(4-methoxyphenyl)-21H,23H-porphine	$C_{48}H_{38}N_4O_4$
4	5,10,15,20-Tetraphenyl-21H,23H-porphine nickel(II)	$C_{20}H_{12}N_4Ni$
5	5,10,15,20-Tetra(p-hydroxyphenyl)-21H,23H-porphine	$C_{44}H_{30}N_4O_4$
6	Hematoporphyrin IX dihydrochloride	$C_{34}H_{40}Cl_2N_4O_6$
7	Cresol Red	$C_{21}H_{18}O_5S$
8	Methyl Red	$C_{15}H_{12}N_3O_2$
9	Curcumin	$C_{21}H_{20}O_6$

Table S3. Results of the runs test applied to detect the nonlinearity between the CSA database and TVB-N reference in chicken by the APaRPs method.

Target	n_+	n_-	u	μ	σ	z	Conclusion
TVB-N	50	30	50	38.5	4.16	2.88	**Nonlinearity**
